# Draft Genome Sequence of *Lactobacillus crispatus* JCM5810, Which Can Reduce *Campylobacter jejuni* Colonization in Chicken Intestine

**DOI:** 10.1128/genomeA.00255-16

**Published:** 2016-04-14

**Authors:** Jessica Wooten, Xiaoji Liu, Michael J. Miller

**Affiliations:** Food Science and Human Nutrition, University of Illinois at Urbana-Champaign, Urbana, Illinois, USA

## Abstract

We present the 2.05-Mb draft genome sequence of *Lactobacillus crispatus* JCM5810, a chicken intestinal isolate with the ability to reduce *Campylobacter jejuni* colonization in chickens. The genome sequence will provide insights on the probiotic mechanisms of *L. crispatus* JCM5810.

## GENOME ANNOUNCEMENT

Lactobacilli comprise the largest genus of lactic acid bacteria (LAB) and are of interest because some possess probiotic characteristics (1). *Lactobacillus crispatus* JCM5810, originally isolated from chicken feces, has gained increased interest because of its potential probiotic properties *in vitro* and *in vivo*. In particular, the ability of *L. crispatus* JCM5810 to reduce *Campylobacter jejuni* colonization in chicken intestine is of great interest (2). *C. jejuni* is a Gram-negative, spiral-shaped bacterium which is the most common bacterial cause of food-borne illness and diarrheal disease in the United States (3). Consumption of contaminated chicken is one of the most common ways humans contract diseases caused by *C. jejuni* (World Health Organization; http://www.who.int/mediacentre/factsheets/fs255/en). In a study conducted by Neal-McKinney et al. (2), experiments were conducted to evaluate the ability of four *Lactobacillus* strains to reduce colonization of *C. jejuni* in commercial broiler chickens. Of the four strains, *L. crispatus* JCM5810 was the most effective in reducing the number of chickens colonized with *C. jejuni*. In addition, they provided evidence that the production of lactic acid by *L. crispatus* JCM5810 was responsible for this activity. More recently, *in vitro* trials demonstrated that *L. crispatus* JCM5810 was able to coaggregate with *C. jejuni* NCTC 11168 (4), suggesting another potential mechanism for how *L. crispatus* JCM5810 reduces *C. jejuni* colonization in chickens.

To better understand the probiotic potential and its genetic traits, the whole genome of *L. crispatus* JCM5810 has been sequenced. Here, we report the draft genome sequence of *L. crispatus* JCM5810, consisting of 7 scaffolds for a total of 2.05 Mb with a GC content of 36.5%. All sequencing was conducted at the Roy J. Carver Biotechnology Center at the University of Illinois at Urbana-Champaign. Whole-genome sequencing was done using the Illumina MiSeq platform, generating a mate-pair library containing 5,226,355 reads with an average length of 300 bp. Data processing, including adapter trimming, quality control, and *de novo* assembly, was performed using CLC Genomics Workbench version 8.5.1 (CLC bio; https://www.qiagenbioinformatics.com/products/clc-genomics-workbench). The sequencing reads were assembled into 12 contigs with an average length of 170,057. These 12 contigs were further constructed into 7 scaffolds by Sanger sequencing. The final orientation and ordering was assisted using *L. crispatus* ST1 (NC_014106.1) as a reference.

### Nucleotide sequence accession numbers.

This whole-genome shotgun project has been deposited at DDBJ/ENA/GenBank under the accession number LSVK00000000. The version described in this paper is the first version, LSVK00000000.1.
